# Social Cognitive Training Improves Emotional Processing and Reduces Aggressive Attitudes in Ex-combatants

**DOI:** 10.3389/fpsyg.2017.00510

**Published:** 2017-04-06

**Authors:** Sandra Trujillo, Natalia Trujillo, Jose D. Lopez, Diana Gomez, Stella Valencia, Jorge Rendon, David A. Pineda, Mario A. Parra

**Affiliations:** ^1^Grupo de Investigación en Salud Mental, Facultad Nacional de Salud Pública, Universidad de AntioquiaMedellín, Colombia; ^2^Doctoral Program in Psychology, Department of Psychology, Universidad de GranadaGranada, España; ^3^Department of Experimental Psychology, University of GranadaGranada, Spain; ^4^Grupo de Neurociencias, Universidad de AntioquiaMedellin, Colombia; ^5^SISTEMIC, Facultad de Ingeniería, Universidad de AntioquiaMedellín, Colombia; ^6^Department of Cognitive Neuroscience, Maastricht UniversityMaastricht, Netherlands; ^7^Grupo de Neuropsicología y Conducta, Universidad de AntioquiaMedellin, Colombia; ^8^School of Social Sciences, Psychology, Heriot-Watt UniversityEdinburgh, UK; ^9^Cerebro, Cognición y Procesos Sociales, Psicologia, Universidad Autónoma del CaribeBarranquilla, Colombia

**Keywords:** emotional processing, social cognitive training, ex-combatants, aggression, intervention

## Abstract

Emotional processing (EP) is a complex cognitive function necessary to successfully adjust to social environments where we need to interpret and respond to cues that convey threat or reward signals. Ex-combatants have consistently shown atypical EP as well as poor social interactions. Available reintegration programs aim to facilitate the re-adaptation of ex-combatants to their communities. However, they do not incorporate actions to improve EP and to enhance cognitive-emotional regulation. The present study was aimed at evaluating the usefulness of an intervention focused on Social Cognitive Training (SCT), which was designed to equip ex-combatants enrolled in the Social Reintegration Route with EP and social cognition skills. A group of 31 ex-combatants (mean age of 37.2, 29 men) from Colombian illegal armed groups were recruited into this study. Of these, 16 were invited to take part in a SCT and the other continued with the conventional reintegration intervention. Both groups underwent 12 training sessions in a period 12–14 weeks. They were assessed with a comprehensive protocol which included Psychosocial, Behavioral, and Emotion Processing instruments. The scores on these instruments prior to and after the intervention were compared within and between groups. Both groups were matched at baseline. Ex-combatants receiving the SCT experienced significant improvements in EP and a reduction in aggressive attitudes, effects not observed in those continuing the conventional reintegration intervention. This is the first study that achieves such outcomes in such a population using SCT intervention. We discuss the implications of such results toward better social reintegration strategies.

## Introduction

Emotional processing (EP) is a broad concept that comprises the ability to perceptually analyze the emotional valence of incoming stimuli, to regulate our self-expression to emotions, and to recognize the emotional state of others (Brand et al., 2016). EP is necessary to successfully adjust to social environments where we constantly need to read, interpret, and act upon cues that can convey either threat or reward signals (Lang et al., 2013). In support to this notion, the recognition of basic features (i.e., perceptual) carrying primary emotional information has been considered a fast and automatic process (Pratto and John, 1991; Öhman et al., 2001) responsible for programing and executing social responses (Fazio and Olson, 2003). This view has emerged from the use of experimental tasks designed to evaluate emotion recognition of faces (Heuer et al., 2007; Hurtado et al., 2009; Luo et al., 2010; Ibáñez et al., 2011, 2014; Petroni et al., 2011; Zhang et al., 2014) or words (Schacht and Sommer, 2009; Ibáñez et al., 2014). Petroni et al. (2011) found that the recognition of face valence is associated to the ability to read others' intentions. EP has also been linked to responses during social conflicts (Seehausen et al., 2012, 2014). Taken together this evidence suggests that EP is crucial for rapidly and accurately scanning the environment in the search of cues that can trigger adaptive social responses.

Ex-combatants have shown atypical EP expressions (Boxer et al., 2011; Tobon et al., 2015; Quintero-Zea et al., 2017). They present with an increased reactivity to emotional images (i.e., International Affective Picture System—IAPS) revealed via late electrophysiological responses that are associated to a reduction in their empathic disposition (Tobon et al., 2015). A more recent study has confirmed that modulations of Event Related Potentials elicited during the EP of faces or words together with the analysis of aggressive responses and social interactions can distinguish between ex-combatants and controls (Quintero-Zea et al., 2017). Ex-combatants are characterized by persistent aggressive behaviors, reduction of moral standards, the presence of mental health problems, impairments in social interactions (Engen, 2008), and dehumanizing tendencies toward their enemies (Williams et al., 2006). Based on these studies, it seems adequate to suggest that the characterization and improvement of EP in ex-combatants will be a crucial step toward identifying routes to enhance their ability to better cope and positively interact with social challenges in post-war conflicts.

Social Cognitive Training (SCT) is a cost-effective, adaptable, evidence-based model that have been used for the intervention of social cognition in schizophrenia and autism related disorders (Turner-Brown et al., 2008; Kurtz and Richardson, 2011; Peyroux and Franck, 2014; Kurtz et al., 2016). It has yielded improvements of basic EP (i.e., face recognition; Kurtz and Richardson, 2011; Kandalaft et al., 2013), as well as of theory of mind and social interaction skills (Turner-Brown et al., 2008; Kandalaft et al., 2013). Similar approaches have proved valid in individuals with Traumatic Stress Disorder (PTSD; Foa, 1997; Foa et al., 2005; Sin and Lyubomirsky, 2009; Akbarian et al., 2015), and also in active military (Castro et al., 2012; Cacioppo et al., 2015) and ex-combatants (Karlin et al., 2010). SCT seems to be a feasible approach to improve emotional recognition in ex-combatants (Kurtz and Richardson, 2011; Tobon et al., 2015; Quintero-Zea et al., 2017). Furthermore, SCT interventions that combine strategies to enhance cognitive-emotional regulation and social-cognition skills may be more effective at improving EP in ex-combatants in a socially meaningful way.

Colombia offers a suitable scenario to investigate this hypothesis. The country has hosted one of the longest war conflicts built mainly on left political ideologies (Dennis, 2006). Amnesty International estimates that, in the past 20 years, more than 70,000 people have been injured or killed and thousands have been kidnapped, tortured, or forcibly abducted to serve in one of the armed forces (Theidon, 2007). The psychological aftermath of war has been reported by civilians after a long-term exposure to violence, by victims of the conflict, as well as by ex-combatants (Nussio and Oppenheim, 2013). Colombia currently undergoes a process of transition to post-conflict via the implementation of a Demobilization, Disarmament and Reintegration (DDR) Program. The social reintegration component of the DDR Program focuses on promoting psychosocial well-being and improving everyday behaviors. However, the evidence supporting its efficacy to enhance cognitive-emotional regulation is currently lacking (Betancourt et al., 2010). Based on a quasi-experimental design, the present study was aimed at investigating whether a SCT intervention adapted for ex-combatants enrolled in the *Reintegration Route* could positively impact on their EP and by this means enhance their cognitive-emotional regulation. We hypothesized that positive outcomes would be observed in the group under the newly devised SCT program but not in the group receiving conventional intervention, which does not target such socio-emotional skills.

## Methods

### Participants

**Figure 1:**
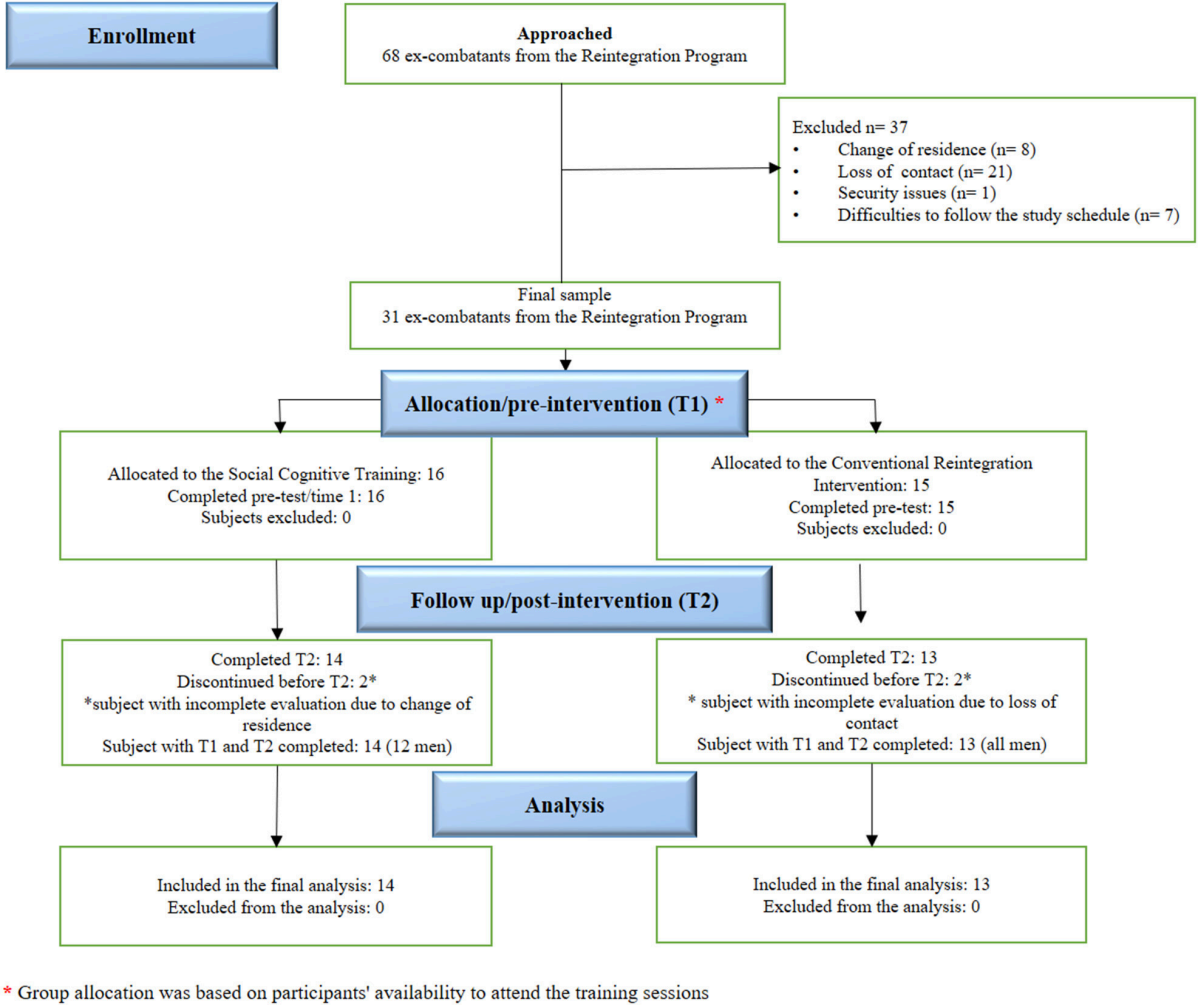
**CONSORT flow diagram illustrating the steps followed during the study**.

Participants were recruited from “Agencia Colombiana para la Reintegración” (ACR; Colombian Agency for Reintegration: http://www.reintegracion.gov.co/en). The ACR is a governmental institution aimed at facilitating psychosocial support (i.e., living costs, occupational education, psychological group activates) toward the re-adaptation of ex-combatants to their communities. After returning to civil life, ex-combatants are offered a set of activities which are part of the “*Reintegration Route Program*: …*a path that each person, in the process led by the ACR must walk through in order to fully reintegrate into the social and economic life…*^1^.” This consists of compulsory (i.e., weekly communication with the route mentor) and optional activities (e.g., baking workshop). Subjects embarked on the *Reintegration Route* are normally enrolled on this program for ~2.5 years (Henao Álvarez, 2013). The total sample comprised 31 subjects (29 men, 29 right handed) aged between 27 and 57 (*M* = 37.16, *SD* = 8.30) and with an average education of 10.23 years (*SD* = 3.03). The sample was divided in two groups. The first group, which we labeled the Social Cognitive Training Intervention Group (SCTIG), involved 16 subjects (14 men and 2 women). The second group, labeled Conventional Reintegration Group (CRG), involved 15 subjects (all were men). The two groups completed the pre-intervention assessment at time 1 (T1). Four subjects, two assigned to the SCTIG and two to the CRG, abandoned the study and did not provide follow up data at time 2 (T2) data. This assessment was performed by two blind trained psychologists who were involved neither in the recruitment nor in the intervention sessions. For the post-intervention assessment the SCTIG and CRG retained 14 (12 men) and 13 (all men) subjects, respectively. Due to limitations to accessing, retaining, and following up individuals from this population, we relied on a Convenience Sampling approach that focused on subjects' availability. Participants enrolled in the *Reintegration Route Program* offered by the ACR were approached and invited to take part in the study. Once they accepted they were allocated to one of the two groups (for details see Figure 1 CONSORT flowchart). The allocation was based on the researchers' decisions, which focused on the availability of the participant to take part in the individual weekly sessions. No personal data or background information was used during the allocation process. Neither the participants' motivation nor their preferences were considered during the allocation process. Participants were excluded if they had a history of present or past use of psychoactive drugs. The sample recruited into this study was free of psychiatric, neurological, or drug related disorders and had not been previously treated due to any these conditions. SCTIG and CRG did not significantly differ in age, gender, or education. Demographic data and descriptive statistics are presented in Table 1.

**Table 1:** **Descriptive statistics for demographic and emotion processing variables, and results from ANOVA models at T1 and at T2 compared to T1 across groups**.

**Variable**	**Time 1**		**Time 2**		**ANOVA T1 (*N* = 31, *df* = 2.27)**	**ANOVA T1 vs. T2 (*N* = 27, *df* = 2.26)**
**Behavioral**	**SCTIG *M* (*SD*)**	**CRG *M* (*SD*)**	**SCTIG *M* (*SD*)**	**CRG *M* (*SD*)**	**Condition × group *F*_(**p**)_, η^2^, β *M* (*SD*)**	**Time × condition × group *F*_(**p**)_, η^2^, β *M* (*SD*)**
**DEMOGRAPHIC**						
Age	39.5(8.18)	35.2 (7.93)	–	**–**	1.45 (0.16)	**–**
Gender (F:M)	2:14	15	–	**–**	0.41 (0.52)	**–**
School level	10.4 (2.91)	10.1 (3.25)	–	**–**	0.27 (0.79)	**–**
**EMOTION PROCESSING ACCURACY**						
F Happy	81.67 (19.89)	83.81 (18.18)	85.56 (22.00)	92.02 (19.03)	2.82 (0.07), 0.3, 0.53	***5.46 (0.01), 0.41, 0.82***
F Neutral	49.69 (28.18)	66.79 (25.56)	65.00 (21.42)	62.86 (26.22)		
F Angry	63.65 (21.99)	62.26 (21.59)	67.33 (25.68)	73.45 (17.15)		
W Pleasant	67.80 (26.48)	65.74 (19.79)	74.46 (21.16)	66.22 (27.54)	0.65 (0.53); 0.15, 0.15	0.21 (0.81), 0.09, 0.08
W Neutral	48.36 (25.98)	57.03 (29.89)	60.44 (25.62)	61.36 (30.03)		
W Unpleasant	63.44 (30.29)	62.86 (23.96)	74.00 (23.63)	72.26 (19.87)		
**REACTION TIME (MS)**						
F Happy	878.90 (172.52)	950.03 (424.18)	968.34 (272.68)	886.51 (284.65)	2.01 (0.14); 0.26, 0.40	0.33 (0.72), 0.11, 0.10
F Neutral	1049.52 (269.59)	906.33 (245.90)	1180.94 (294.73)	983.68 (224.49)		
F Angry	945.18 (159.74)	1024.46 (348.79)	1101.10 (236.81)	1000.08 (303.68)		
W Pleasant	1107.77 (410.59)	1027.85 (305.60)	1175.04 (310.97)	1114.77 (337.66)	1.37 (0.35); 0.19, 0.23	1.25 (0.30), 0.21, 0.26
W Neutral	1243.32 (567.11)	1056.47 (337.06)	1176.70 (263.71)	1032.58 (300.42)		
W Unpleasant	1191.13 (525.67)	1150.78 (399.25)	1257.35 (272.19)	1101.10 (341.42)		
**Error type (%)**						**Time × Error Type × group *F*_(**p**)_, η^2^, β**
Happy err. Neutral	8.0 (10.39)	4.52 (6.45)	8.78 (17.61)	2.02 (2.55)	1.11(0.30), 0.02, 0.17	0.09 (0.77), 0.05, 0.06
Happy err. Angry	10.11 (11.84)	11.67 (15.99)	5.67 (8.49)	5.95 (18.03)		
Neutral err. Happy	20.83 (17.95)	17.14 (15.21)	14.44 (11.33)	14.88 (14.46)	0.2 (0.67), 0.08, 0.07	0.72 (0.41), 0.17, 0.13
Neutral err. Angry	22.52 (11.78)	16.07 (13.25)	20.56 (12.75)	22.26 (18.1)		
Angry err. Neutral	11.44 (16.49)	11.55 (13.63)	8.67 (8.89)	5.24 (6.66)	0.01(0.92), 0.0, 0.05	0.02 (0.88), 0.03, 0.05
Angry err. Happy	25 (15.58)	26.07 (19.52)	24 (18.35)	21.31 (11.59)		
Pleasant err. W neutral	14.35 (9.26)	19.74 (14.42)	14.58 (13.37)	19.85 (13.79)	0.43 (0.52), 0.13, 0.10	0.10 (0.76), 0.02, 0.06
Pleasant *err*. Unpleasant	13.56 (14.73)	14.53 (15.34)	10.96 (14.03)	13.92 (21.39)		
Wneutral err. Pleasant	36.27 (10.28)	25.14 (13.83)	29.18 (15.01)	27.05 (19.17)	1.79 (0.19), 0.25, 0.25	0.34 (0.57), 0.11, 0.09
Wneutral er. Unpleasant	14.92 (14.83)	11.01 (11.32)	10.38 (12.8)	11.59 (15.72)		
Unpleasant err. Pleasant	15.66 (18.73)	20.24 (16.70)	12.89 (15.41)	10.6 (10.91)	0.32(0.58), 0.11, 0.08	1.67 (0.21), 0.24, 0.24
Unpleasant err. Neutral	17 (16.62)	16.90 (15.54)	13.11 (11.53)	17.14 (14.42)		

*F, face condition; W, word condition; SCTIG, Social Cognitive Training Intervention Group; CRG, conventional reintegration group; Err, error; Wneutral, neutral word. Results that reached the significance threshold are presented in italic and bold*.

During the first session, participants were individually informed about the characteristics of the initial and final assessments, and about the structure of the 12-session intervention program. All the participants read and signed the informed consent before starting the study. The study's procedures and informed consent were approved by the Bioethical Committee of the Faculty of Medicine from University of Antioquia, Medellin, Colombia. Participants were requested to confirm their availability to attend 1 h-per-week session at the University of Antioquia for 12 weeks.

### Assessment protocol

The assessment included measures of social and behavioral responses to aggressive experiences and an experimental task that assesses cognitive aspects of EP.

### Emotion processing instrument

*The Emotion Recognition Task* (ERT) is a face and word recognition task adapted from previous studies (Hurtado et al., 2009; Ibáñez et al., 2010, 2011; Petroni et al., 2011). This cognitive function has been thought of as a building block of social cognition (Green et al., 2008; Kurtz and Richardson, 2011; Pinkham et al., 2014). In the context of our study, we interpret the outcomes from such a task as evidence of cognitive functioning that can be crucial to support core aspects of social cognition. We implemented a version of this task in E-prime (Psychology Software Tools, Pittsburg, USA). The task was divided in two blocks, each comprising 90 stimuli. Of these 45 were faces and 45 were words (Faces: 15 happy, 15 neutral, and 15 angry; Words: 15 pleasant, 15 neutral, and 15 unpleasant). Pictures of female and male faces taken from the MMI Facial Expression Database were used in this task (Pantic et al., 2005). Words were selected from the linguistic corpus generated by the communications faculty of the Universidad de Antioquia (Grajales Alzate, 2011) which offers a list of the most commonly used words in this region of Colombia. The stimulus (word or face) was presented on a 17″ screen, placed 60 cm away from the participant's eyes. Each stimulus was presented twice within the same block and there were no more than two consecutive stimuli presenting the same valence.

**Figure 2:**
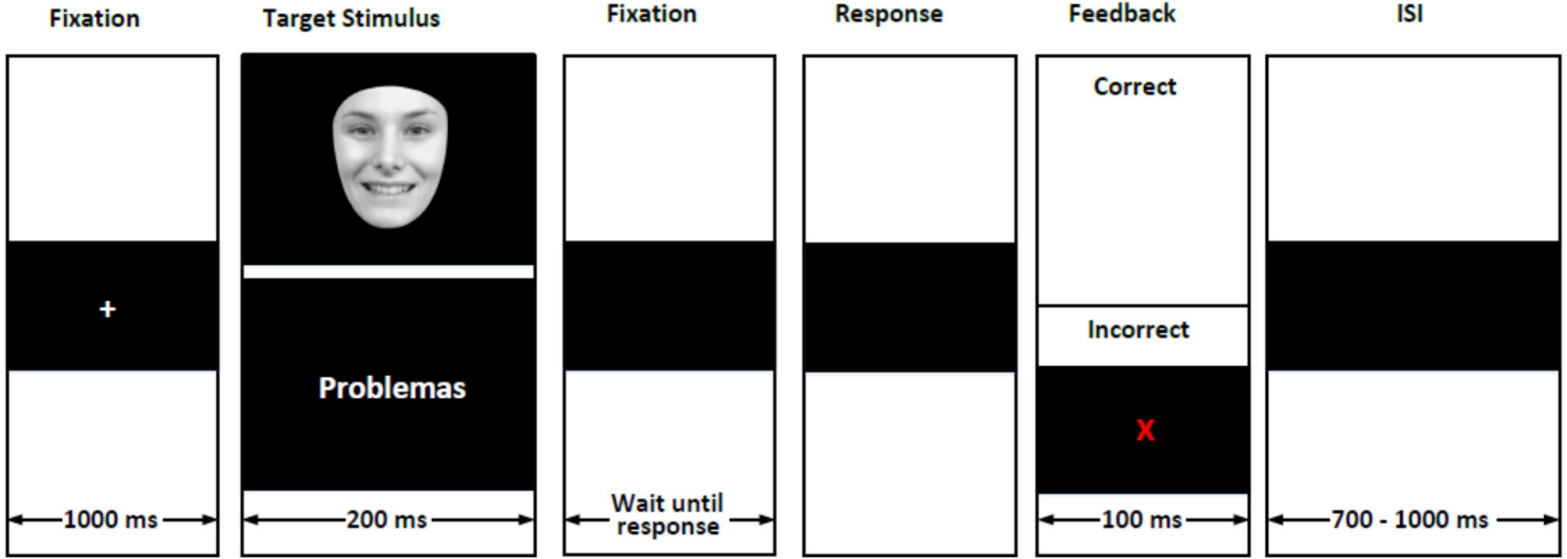
**Sequence of the emotion recognition task for both stimulus categories: faces and words**.

The task sequence is shown in Figure 2. A fixation cross was presented for 1,000 ms which was followed by the stimulus display (i.e., face or word) presented for 200 ms. Immediately after, the participants' response was requested. If the stimulus was a face, they were asked to decide whether it showed a happy, neutral, or angry expression. If the stimulus was a word, they were asked to decide whether it described a pleasant, neutral, or unpleasant emotion. Participants entered their responses by pressing one of three keys previously allocated of a standard PC keyboard. Correct responses were followed by a black screen which appeared for a random duration between 700 and 1,000 ms (i.e., inter trial interval). Incorrect response were indicated by a red letter “X” which appeared in the center of the screen for 100 ms. This feedback was used to encourage attention to the task. The feedback screen was followed by the inter trial interval described above. We recorded reaction time, the number of hits, and the errors. The ERT has been previously used in SCT intervention studies involving adults with high functioning autism (Turner-Brown et al., 2008); schizophrenia (Kurtz and Richardson, 2011), and also in the assessment of Colombian ex-combatants (Quintero-Zea et al., 2017).

### Psychosocial and behavioral instruments

*Situation and Aggressive Behavior Inventory* (ISCA: Spanish acronym; Juarez-Acosta and Montejo-Hernandez, 2008). The instrument assesses the expression of violent behaviors triggered by different situations during the last 4 weeks prior to assessment. It is divided into two subscales: *situations* with 13 items and *behaviors* with 13 items. Participants responded using a 3-point Likert scale (1 = never, 2 = sometimes, 3 = often) and received a separate score for each subscale and a global score. ISCA has an alpha of Chronbach of 0.87 and 0.81 for each respective subscale, and 0.79 for the global score.

*Motives for Aggression Inventory* (IMA: Spanish acronym; Juarez-Acosta and Montejo-Hernandez, 2008) is a self-report scale comprising 26 items rated on a 3-point Likert scale (1 = *never/almost never*, 2 = *sometimes*, and 3 = *frequently*), indicating frequency of each motive leading to aggressive behaviors. The scale asks questions such as “*When you act aggressively, is because you feel too tense?*” This questionnaire is based on the concept that violent behaviors vary as a function of the intensity of their drivers. It yields a global score which has shown an alpha of Cronbach of 0.91 (Juarez-Acosta and Montejo-Hernandez, 2008).

*Interpersonal Reactivity Index* (Davis, 1983) assesses dispositional empathy and sensitivity to experiences of others. The Spanish version was adapted by Escrivá et al. (2004). The scale has 28 self-report items of which 19 are redacted in a positive sense and 9 in a negative sense. Responses are entered using a 5-point Likert scale (1 = *it does not describe me well* to 5 = *it describes me very well*). The scale is divided in four dimensions: *Perspective Taking* (PT), *Empathic Concern* (EC), *Fantasy* (FS), and *Personal Distress* (PD). PT evaluates the ability to consider other's points of view. EC assesses the response to feelings of compassion or sympathy through recognizing others' misfortunes. FS explores the ability to self-identify as a fictional character in a story such as novels, books, or movies. PD measures self-oriented negative arousal in response to stressors, attitudes, and experiences of other people. The reliability of the scale ranges from 0.70 to 0.77 (Escrivá et al., 2004). The instrument was standardized for Colombian ex-combatants (Pineda et al., 2013; Garcia-Barrera et al., 2017).

*Social Skills (SS) Scale* (Gismero, 2000) is a self-report instrument that evaluates everyday social behaviors via 33 items. This scale enquires individuals about their ability to interact with others in different situations. Items are grouped in six dimensions: *(1) self-expression in social situations, (2) defense of own rights as a consumer, (3) expression of anger or displeasure, (4) stop interactions and saying no, (5) make requests, (6) start positive interactions with the opposite gender*. Responses are recorded using a 4-point Likert scale (1 = *I do not identify with that at all/most of the time it does not happen/I would not do it* to 4 = *I totally agree/most of the time/I would behave like that*). The scale has an alpha Cronbach of 0.88 and has demonstrated to be sensitive to SS variations in normal population (Gismero, 2000). In this study, we focused on the Global SS Score. Larger values of this score suggest reduced social assertion.

### Intervention programs

#### Social cognitive training intervention group (SCTIG)

The SCT was a low-intensity, brief (45 min, 12 sessions) individual intervention, developed for the purpose of this study, to be used in former combatants from Colombian illegal armed groups. The intervention aimed to improve social skills, theory of mind as well as EP (Kurtz and Richardson, 2011). Sessions of the SCT intervention involved (a) the discussion of the subject's response in hypothetical social interactions, (b) social scene simulations such as role playing, (c) revisiting individual response on everyday situation, and (d) performance of tasks which require applying skills developed via the new training (Kurtz and Richardson, 2011; Kandalaft et al., 2013; Peyroux and Franck, 2014). The SCT consisted of the following axes: *Axis 1* focused on the identification of basic emotions (session 1–3) emphasizing on the improvement of emotional recognition skills. *Axis 2* focused on social skills and assertive expressions of emotion in everyday situations (session 4–8). *Axis 3* enhanced aspects of theory of mind and social-cues reading (session 9–12).

This program was designed to train individuals to accurately recognize and interpret social cues as well as basic and complex emotions. For example, in *Axis 1*, participants learned about the role of basic emotions and associated cues (Happy, Anger, Disgust, Fear, Surprise, and Sadness) and how to recognize such emotions in themselves and in others relying on imaginary or real-life situations. After each session, the therapist recommended additional work to do with their families and co-workers outside the training context which encouraged ex-combatants to further apply the acquired knowledge to daily living scenarios. The therapist requested inputs from such additional work and provided feedback. We anticipated that such a program would (re)equip ex-combatants with the skills needed to identify the intentions of others in everyday situations and use assertiveness to manage aggressive responses.

#### Conventional reintegration group (CRG)

The CRG took part in a 45 min weekly session which were aimed at developing competencies in family life, education, work, community challenges, and problem solving. As we mentioned before, ex-combatants are normally embarked on the *Reintegration Route Program* for ~2.5 years (Henao Álvarez, 2013). However, the time each individual spends in the *Reintegration Route Program* might be tailored by the ACR according to factors only known to them (ACR keeps this information strictly confidential). Educational achievements and improvements in daily life performance are monitored monthly whereas work adaptation and community participation are assessed every 6 months. These data are kept in strict confidentiality by the ACR.

### Procedures

**Figure 3:**
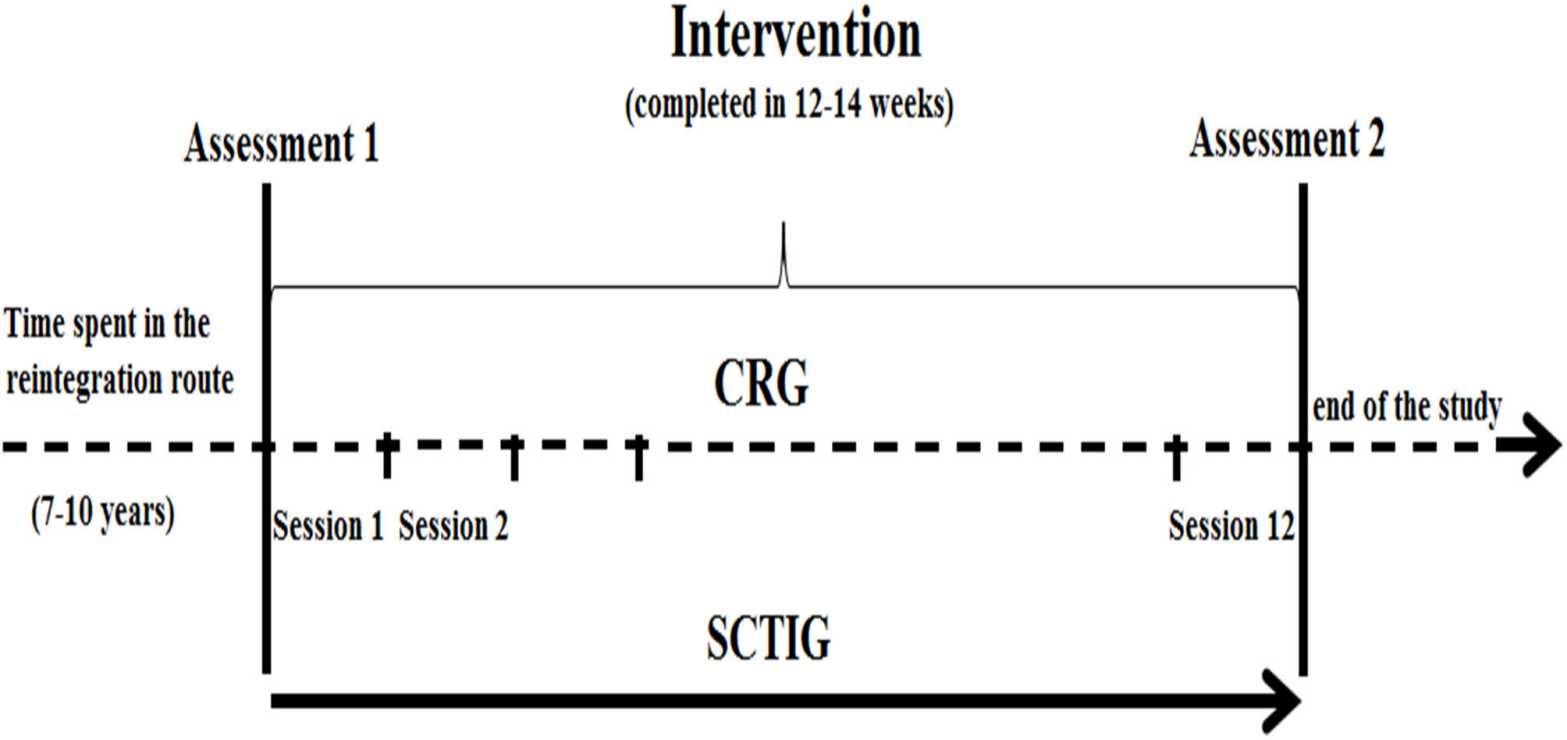
**Diagram illustrating the study design**.

The study followed a pre-post intervention design which includes a comprehensive assessment protocol comprising cognitive and psychosocial-behavioral instruments that were applied prior to and after two types of interventions. After consenting participants were given a set of questionnaires and tests to gather baseline data (T1). They then went to receive the newly devised intervention program (SCTIG group) or to continue with the standard intervention program offered by the ARC (CRG group). After completing 12 intervention sessions the same assessment protocol was applied (T2). This assessment was performed by two blind trained psychologists who were involved neither in the recruitment nor in the intervention sessions. The cognitive and psychosocial-behavioral components of the assessment were counterbalanced across participants and the same counterbalancing order was used during the pre- and post-intervention assessment. The SCT applied to the SCTIG was always delivered by the same psychotherapist. The psychotherapist was a psychologist with advanced clinical training and expertise in the intervention of similar populations. Ex-combatants allocated to the SCTIG group stopped attending the regular training offered by the ACR for the duration of the study. The CRG continued attending the regular training delivered by the professional staff from the ACR and was the only intervention they received. Due to confidentiality issues, the exact time each participant had spent in the *Reintegration Route Program* was not disclosed to the research team. All the participants however, were part of the demobilization agreement derived from the Peace and Justice Law from 2003 to 2006. By the time of this study, they should have been enrolled in the *Reintegration Route Program* from 7 to 10 years. Each assessment was separated by a window of 12–14 weeks which was filled with the intervention programs (see a diagram of the study design in Figure 3). The duration of each assessment session was approximately of 2.5 h per participant.

### Statistical analysis

Demographic variables were compared using independent sample *t-*tests or χ^2^. Psychosocial and behavioral variables drawn from assessment 1 (T1) were compared across SCTIG and CRG using independent-sample *t*-tests. This analysis was aimed at ascertaining that there were no baseline differences in psychosocial scores across the investigated groups. To investigate the effect of the intervention we implemented a mixed ANOVA model which included Group (SCTIG vs. CRG) as a between-subjects factor and Time (T1 vs. T2) as the repeated measure. Our outcome measures for psychological rating scale were: IMA, ISCA-situation, ISCA-conduct, IRI perspective taken; IRI fantasy; IRI empathic concern; IRI personal distress; Social skills. When necessary, we ran ANCOVA to control for potential confounders.

For the ERT, our outcome measures were the percentage of correct responses (accuracy), reaction time and error type (see definition below) across each emotional Condition (Face: happy, neutral, or angry; Word: pleasant, neutral, or unpleasant). Mixed ANOVA models were implemented for the mean reaction time and accuracy on each Time (T1 vs. T2) and for each Condition (Face: happy, neutral, or angry; Word: pleasant, neutral, or unpleasant) across Groups (SCTIG vs. CRG). Errors were calculated as the proportion of responses within each alternative valence (i.e., type) erroneously assigned to the to-be-judged valence. For example, if the stimulus presented a Neutral face, two types of errors could be committed i.e., Neutral-Happy whereby the subject identifies a happy emotion or Neutral-Angry whereby the subject identifies an angry emotion. The analysis of Error Type across task conditions is relevant as it would inform whether poor accuracy is driven by a particular bias toward a specific emotion and whether the pattern of bias differs across groups. The ANOVA model to analyze Error Type was similar to that above described but included this new additional repeated measure. For main effects and interactions effect size was informed by eta-square (η^2^) (0.1 = small, 0.24 = medium, and 0.31 = large; Cohen, 1988) and power by β. To further explore significant interactions, Tukey corrected *post-hoc* analyses were carried out. For these contrasts, the effect size was calculated using the Cohen's d (0.2 = small, 0.5 = medium, and 0.8 large; Cohen, 1973). For significant 3-way interactions we calculated the T2-T1 discrepancy for each relevant variable and entered these values to *post-hoc* tests. Those discrepancies that reached the significance threshold in ANOVA models were subjected to stepwise linear regression models. These models were implemented to identify the predictive values of those variables yielding significant effects in the previous analysis. To this aim, we selected cognitive variables from the ERT (i.e., behavioral: Accuracy/Error Type/Reaction Time) which were included as dependent variables and psychosocial-behavioral variables from rating scales that identified between-group differences in previous analyses which were entered as predictors. All the analyses were performed in IBM SPSS 21 for windows.

## Results

### Emotion processing assessment

The analysis of demographic variables showed no significant differences between SCTIG and CRG in age, gender, or education (Table 1). Table 1 also presents the mean and standard deviation for accuracy, reaction time, and error type at T1 and T2 for each group as well as the outcomes from the mixed ANOVA models. The first model assessed group differences at baseline (Condition × Group) and the second the effects of the intervention (Time × Condition × Group). For the sake of brevity, we report only on the key interactions (i.e., 3-way interactions). Baseline models revealed no significant effects thus confirming that the two groups could not be differentiated based on baseline measures of emotion processing.

ANOVA models investigating the effects of the intervention on Face processing using accuracy data revealed a significant Time by Condition by Group interaction [*F*_(1, 27)_ = 5.46, *p* = 0.01, η^2^ = 0.41, β = 0.82]. *Post-hoc* contrasts were carried out across conditions for each group separately entering differences between T2 and T1 (see Section Statistical Analysis above). SCTIG ex-combatants showed a significantly larger discrepancy in accuracy for neutral faces (T1 < T2) than for happy faces (T1 = T2) (*t* = 2.37, *p* = 0.03, *d* = 0.85). No other contrasts revealed significant differences. No significant differences were observed for CRG ex-combatants. *Post-hoc* contrasts carried out across groups for each condition separately also entering differences between T2 and T1 revealed a significant discrepancy (T1 < T2) for SCTIG ex-combatants in accuracy for neutral faces (*t* = 3, *p* = 0.01, *d* = 1.12) an effect not observed in the CRG group. No other contrasts revealed significant differences. ANOVA models investigating the effects of the intervention on Word processing using accuracy data failed to reach the significance threshold for the 3-way interaction [Time by Condition by Group: *F*_(1, 27)_ = 0.21, *p* = 0.81, η^2^ = 0.09, β = 0.08].

ANOVA models investigating the effects of the intervention on Face and Word processing revealed significant 3-way interactions neither for Reaction Time data nor for the Type of Error committed (See Table 1).

### Psychosocial and behavioral scales

Between-group comparisons of outcomes from Psychosocial scales applied during the pre-intervention assessment (Time 1) revealed significant differences on ISCA behavior whereby the CRG group showed higher score than the SCTIG group. However, these scores did not yield a significant interaction in the ANOVA model (see Table 2). Thus as for emotion processing, the two groups could not be differentiated based on baseline outcomes from the Psychosocial and Behavioral rating scales. For the *Motives for Aggression Inventory* (IMA), the ANOVA model identified a significant Time by Group interaction. This was driven by lower scores of the SCTIG group at T2 relative to T1, an effect not observed in the CRG group (see Table 2). Such an interaction was still present after correcting for ISCA (i.e., ANCOVA) [*F*_(1, 27)_ = 12.86, *p* < 0.001, η^2^ = 0.57, β = 0.93].

**Table 2:** **Descriptive statistics for variables from the psychosocial and behavioral rating scales, and results from ANOVA models at T1 and at T2 compared to T1 across groups**.

**Variable**	**Time 1**		**Time 2**		***t*-test at T1 (*N* = 31)**	**ANOVA T1 vs. T2 (*N* = 27, *df* = 1)**
	**SCTIG *M* (*SD*)**	**CRG *M* (*SD*)**	**SCTIG *M* (*SD*)**	**CRG *M* (*SD*)**	**SCTIG vs. CRG *t*_(**p**)_**	**Time × group *F*_(**p**)_, η^2^, β**
**DEMOGRAPHIC**						
Age	39.5(8.18)	35.2 (7.93)	–	–	1.45 (0.16)	–
Gender (F:M)	2:14	15	–	–	0.41 (0.52)	–
School level	10.4 (2.91)	10.1 (3.25)	–	–	0.27 (0.79)	–
**PSYCHOSOCIAL**						
IMA	38.13 (10.84)	33.86 (7.18)	27.94 (8.91)	34.57 (11.0)	1.25 (0.22)	***6.25 (0.02), 0.42, 0.68***
ISCA1	27.38 (3.20)	27.71 (4.55)	13.88 (4.62)	16.36 (3.75)	0.24 (0.81)	0.97 (0.33), 0.18, 0.16
ISCA2	10.06 (1.12)	11.14 (1.51)	8.94 (2.54)	10.71 (1.77)	***2.24 (0.03)***	0.49 (0.49), 0.41, 0.10
IRIPT	16.38 (5.38)	17.50 (4.50)	17.13 (3.94)	18.92 (4.70)	–0.62 (0.54)	0.18 (0.68), 0.08, 0.07
IRIF	13.69 (4.76)	12.86 (4.74)	11.47 (5.38)	12.62 (5.81)	0.48 (0.64)	0.58 (0.45), 0.15, 0.11
IRIEC	13.81 (5.42)	14.14 (3.46)	18.80 (4.31)	19.92 (5.09)	0.20 (0.85)	0.06 (0.81), 0.05, 0.06
IRIPD	11.81 (5.13)	11.38 (4.33)	10.20 (4.48)	9.62 (4.25)	0.66 (0.51)	0.00 (0.98), 0.00, 0.05
SSG	71.31 (15.10)	64.43 (16.46)	68.23 (21.68)	64.00 (34.8)	1.19 (0.24)	0.09 (0.77), 0.06, 0.06

*IMA, Motives for Aggression Inventory; ISCA, Situation and Aggressive Behavior Inventory; ISCA1, ISCA-situation dimension; ISCA2, ISCA-conduct dimension; IRI, interpersonal reactivity index; IRI PT, perspective taken; IRI FS, fantasy; IRI EC, empathic concern; IRI PD, personal distress; GSS, Global social skills score; SCTIG, Social Cognitive Training Intervention Group; CRG, Conventional Reintegration Group; T1, time 1; T2, time 2. Results that reached the significance threshold are presented in italic and bold*.

In sum, the analysis of Emotion Processing (i.e., ERT) and Psychosocial and Behavioral Rating Scales revealed an impact of the intervention program which was characterized by an increase in accuracy during the recognition of neutral faces, as informed by the former assessment, and a reduction of aggressive attitudes, as informed by the latter test. These effects were observed in SCTIG ex-combatants only.

Finally, accuracy data from the ERT that yielded significant effects in the ANOVA model and that from the Psychosocial and Behavioral Rating Scales entered regression analysis. This was aimed at investigating potential associations between behavioral and psychosocial improvements resulting from the intervention. A stepwise regression analysis with Accuracy fixed as the dependent variable and Aggression IMA score as the predictor showed a significant association [*B* = −0.53; *F*_(1, 29)_ = 10.41, *p* < 0.001]. Thus, improvements in controlling the influence of aggressive behavior triggers (i.e., informed by lower IMA scores) were associated to improvements in recognizing neutral faces among faces of negative or positive valence (i.e., informed by higher accuracy).

## Discussion

This study was set out to investigate whether a SCT intervention program adapted for ex-combatants enrolled in the *Reintegration Route Program* could positively impact on their EP and in doing so improve their cognitive-emotional regulation. We predicted positive outcomes for SCTIG. Three main findings lend support to this hypothesis. The SCT delivered to SCTIG ex-combatants (1) significantly improved the recognition of neutral faces and (2) reduced aggressive attitudes, effects not observed in CRG ex-combatants. (3) Enhancement of EP significantly predicted a reduction of aggressive behavior triggers. These findings are discussed in turn.

To date, the literature on EP has focused on the analysis of responses to stimuli conveying emotionally relevant information which are contrasted to neutral stimuli. Interpreting the response to neutral stimuli has not been the focus of such analyses. The influence of neutral stimuli on EP has more recently become a topic of interest. For example, the role of neutral faces on EP has been a controversial topic in the literature on affective neuroscience (Güntekin and Başar, 2014; Camfield et al., 2016; da Silva et al., 2016). While some consider that neutral faces, as a baseline condition in Facial Emotion Recognition tasks, have a passive role (Sprengelmeyer et al., 1998; Kesler-West et al., 2001; Pessoa et al., 2002; Kilts et al., 2003), others suggest that correct neutral categorization is contingent upon a meticulous reading of embedded contextual cues. Such a reading is time consuming whereas for emotional faces the recognition is faster (Vuilleumier and Pourtois, 2007; Foti and Hajcak, 2008; MacNamara et al., 2009, 2011). The results from our study suggest that processing neutral faces is far from being a passive process. In the context of the ERT, processing neutral faces appears to index valence recognition mechanisms necessary to resolve ambiguity and uncertainty, which are pillars of successful social interactions (Harris and Menzies, 1998; Schupp et al., 2004; Vuilleumier, 2005; Vuilleumier and Pourtois, 2007).

In line with previous studies, we showed that changes in EP observed in the SCTIG are likely driven by reorganization of brain areas necessary for perceptual analysis of faces in connection with areas implicated in the attribution of valance (Mazza et al., 2010; Popov et al., 2015; Campos et al., 2016). Using EEG, improvements in alpha power modulations have been observed in schizophrenia patients after training aimed at enhancing the analysis of perceptual features to promote social abilities (Popov et al., 2015). Mazza et al. (2010) also found enhancement in emotional recognition of anger, disgust, and sadness through neuropsychological and physiological (i.e., N200) responses after an intervention relying on imitation theories (Mazza et al., 2010). More recently, a systematic review of studies reporting on neural changes after social cognitive training in patients with schizophrenia, identified increased efficiency of brain areas such as superior temporal lobe, fusiform and middle occipital gyrus, known to be involved in automatic face encoding during emotional processing (Campos et al., 2016). The authors suggested that patients develop visual strategies to serially scan facial traits which in turn improves their ability to identify emotions. Furthermore, functional changes observed in such populations were linked to improvements in behavioral performance (Campos et al., 2016). So, there is enough evidence to suggest that positive outcomes from intervention programs, as the one reported here, do not reflect transient changes which will fade away at the intervention offset (though this is still a contentious point). Instead, they seem to result from the reorganization of core brain functions and networks which may enable such benefits endure future social challenges. Future studies should investigate the long-term persistence of such benefits and their role as predictors of successful social re-insertion.

The SCTIG received an intervention that incorporates actions toward the improvement of abilities necessary to infer others' mental state via facial expressions, a function that is essential for social interactions (Ekman and Friesen, 1971; Ekman, 1992; Shariff and Tracy, 2011). It is plausible to suggest that SCT boosted SCTIG ex-combatants' skills to discriminate between emotional valences and absence of emotion (i.e., neutrality; e.g., Baudouin et al., 2000; D'Argembeau et al., 2003; Savaskan et al., 2007; Liu et al., 2015). People enrolled in chronic armed conflicts are more accustomed to constantly experience strong emotions than absence of emotion. From this perspective, recognition of neutral stimuli may pose a greater challenge to ex-combatants than recognition of negative or positive stimuli. Before the SCT, they tended to miss-categorize neutral faces without a preferential pattern, as shown by the Error Type analysis. This ability significantly improved only after the SCT received by the SCTIG. Hence, the SCT seems to have promoted not just the recognition of emotions but the ability to resolve discrepancies in the identification of emotions.

A second relevant finding of our study was a significant reduction of aggressive attitudes in the SCTIG. Previous studies have reported high levels of aggressive behaviors in ex-combatants (e.g., Rona et al., 2015). Former combatants have shown high scores on the Appetitive Aggression Scale (Köbach et al., 2015). Higher scores on this scale are linked to an increased risk to get involved in physical confrontations, relational violence, difficulties to regulate anger (Orcutt et al., 2003; Shea et al., 2013), and larger prevalence of externalized conduct disorder (Elbogen et al., 2014; Sherman et al., 2015). Using automatic classification algorithms (i.e., Support Vector Machine), Quintero-Zea et al. (2017) recently found that proactive (i.e., instrumental) and reactive (i.e., impulsive) aggression scores segregated Colombian ex-combatants from controls. By relying on psycho-emotional strategies that equipped ex-combatants with knowledge about everyday challenges and skills to deal with them, the SCT reduced the influence of everyday triggers that typically elicit aggressive reactions. Similar effects have been observed in individuals with PTSD, personality disorders, eating disorders, autism, and disruptive behavior disorders after psycho-educational interventions (Lukens and Mcfarlane, 2006).

Our third and key finding was an association between improvements in EP and reductions in aggressive attitudes as a result of the SCT. Better EP is known to facilitate self-regulation (Weierstall et al., 2013). For example, improvements in the recognition of neutral faces have been found to be associated with reductions of aggressive drives (Quadflieg et al., 2012; Spisak et al., 2012). Individuals with borderline personality disorders present with a negative bias to neutral faces (Wagner and Linehan, 1999) and also tend to misinterpret interpersonal situations (Veen and Arntz, 2000; Lazarus et al., 2014). Quintero-Zea et al. (2017) reported that in ex-combatants, atypical EP is associated to aggressive responses. Our findings suggest that SCT promotes a better reading of neutral faces and a better interpretation of ambiguous situations thus leading to a reduction in the selection of aggressive responses. Resolving emotional ambiguity may be a skill hampered by a chronic exposure to violent experiences. Whether because of the high frequency of emotionally loaded experiences or because of the few alternatives in terms of response choices, ex-combatants' socio-emotional system seems to bias toward contextually relevant options i.e., aggressive responses to emotionally loaded stimuli. By helping them (re)expand the emotion recognition spectrum along its continuum, and map the links between emotion and response onto everyday life situations, the SCT restored fundamental aspects of EP and social cognition which should enable a smooth social reintegration.

There are some limitations to this study which are worth considering. First, we acknowledge that a proper randomization procedure was not possible in the present study (see Section Methods for a description of our sampling approach). Albeit our efforts to avoid as much as possible potential selection bias, future studies may rely on standard randomization techniques to replicate the findings reported here. Second, although our statistical analyses revealed large effect sizes and acceptable power for key study outcomes, it might still be possible that a small sample size could have precluded the identification of other relevant findings. To overcome this limitation we focused only on results from carefully controlled statistical models which extracted only the most robust evidence. Future studies will be needed to replicate and expand the evidence reported here as well as to evaluate the stability of the benefits drawn from SCT in the long term. Increasing the sample size will also allow identifying differential profiles among ex-combatants (e.g., antisocial personality) what would substantiate the analysis and further identification of bio-psycho-social phenotypes of those leaving the weapons behind and opting for the social *Reintegration Route Program*. It will be also necessary to investigate whether the time elapsed between the demobilization and the initiation of the SCT matters. In this study, both groups had been enrolled in the ACR program for several years. The influence that war experiences exerted on them may have vanished through the effects of the program or time itself. Although this will be worth addressing in future studies, in the context of the present study it can be considered a strength as a randomly selected group of ex-combatants who were given an alternative SCT intervention experienced benefits in areas of EP and social cognition not observed in those who continued the traditional intervention program. This reinforces the notion that innovative solutions which focus on actions that have a meaningful impact on socio-cognitive abilities are of a paramount importance for a faster and more successful post-war conflict reintegration. One final limitation worth mentioning is the identification of psychiatry profiles. The assessment and identification of psychiatry profiles among subjects enrolled in this type of study will allow controlling for potential confounding factors which are likely associated with the presence of mental health disorders such as depression, anxiety, or others.

## Conclusion

A brief SCT program can improve EP and reduce aggressive attitudes in ex-combatants. To our knowledge this is the first study in which SCT intervention focusing on EP is delivered to a sample of ex-combatants embarked on the *Reintegration Route Program*. In addition to unveiling fundamental features of the cognitive (EP) and psychosocial (aggression) phenotype of ex-combatants, we have revealed their association and sensitivity to SCT. The ACR and similar organizations from other countries which also face similar social challenges should consider this alternative intervention as it can equip those who decide to return to society with better psychosocial coping mechanisms.

## Author contributions

ST and NT conceived and designed the study, collected, data and drafted the manuscript. MP and JL conceived and designed the study, and drafted the manuscript. DG, JR, and SV collected data and participated in group discussion and analysis sessions. DP conceived and supervised the study. All the authors agreed on the final version of this manuscript.

## Funding

This work was supported by the Colciencias under Grants (111548925190, 122266140116 and 111556933399). “Fondo primer proyecto—CODI” (INV 518-16) and Newton-Caldas Fund (BC027-EDU2016).

### Conflict of interest statement

The authors declare that the research was conducted in the absence of any commercial or financial relationships that could be construed as a potential conflict of interest.

## Footnotes

^1^http://www.reintegracion.gov.co/en/reintegration/Pages/route.aspx.
